# Beyond good and evil: The pursuit of philosophical and scientific truth in a time of moral ambiguity

**DOI:** 10.1038/s44319-025-00465-x

**Published:** 2025-05-01

**Authors:** G Paolo Dotto

**Affiliations:** Professor of Dermatology, Harvard Medical School, President, International Cancer Prevention Institute (iCPI), Boston, MA USA

**Keywords:** History & Philosophy of Science, Neuroscience

## Abstract

The contributions of philosophy from antiquity to the 20th century and modern neuroscience to understanding human’s capability to commit deeds of great good and great evil.

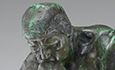

The modern PhD degree originated as a Doctorate in Philosophy in 19th-century Germany but traces its roots back to 12th-century European universities and the Madrasas—institutions of higher education established as early as during the 11th century in the Islamic world. These institutions offered advanced studies in theology, law, science and medicine, emphasizing the interconnected nature of knowledge. Over time, the PhD has become synonymous with specialized expertise from many diverse fields of investigation. Yet, at its core, the PhD should remain true to the essence of its designation: *philosophy*, meaning *love of truth*. Scholars are called to uphold and defend the enduring value of seeking and spelling out the truth, in whatever form it takes. This commitment has a key societal role, helping to address and correct the distorted realities in which we live, uncover forgotten or new knowledge, and inform our decisions accordingly.

… at its core, the PhD should remain true to the essence of its designation: *philosophy*, meaning *love of truth*.

From the earliest days of philosophical inquiry, when human reason took center stage, the pursuit of knowledge and truth (*epistemology*) was deeply intertwined with the principles that guide human action (*ethics*)—in fact, these are two sides of the same coin. In their quest to uncover the ultimate foundation of the universe, early philosophers were also scientists. The Pythagoreans, for instance, combined their fascination with mathematics, geometry, and music with the belief that numerical harmony is key to both physical and mental ‘wholeness’, ideas that intersected with the early teaching of medicine. The Greek philosopher Plato, who held that ultimate Truth and Good can only be approached through approximation, regarded mathematics and geometry as not only vital for personal intellectual development but also essential for the organization and governance of society. However, what is the source of Good and Evil, and is it ever possible to distinguish between them with any degree of certainty (Fig. 1)?

**Figure 1:**
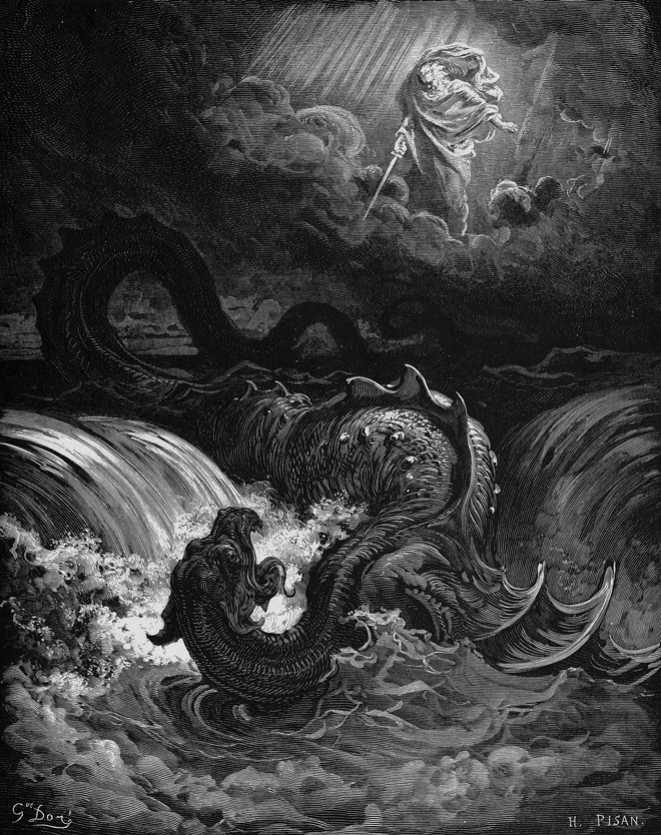
Gustave Dorè (1865). Destruction of Leviathan. Engraving. Wikimedia/Public Domain.

## The quest for truth

*Jenseits von Gut und Böse* (Beyond Good and Evil) is the title of a seminal work by the German philosopher Friedrich Nietzsche, evoking the catastrophic consequences of the nihilism that shaped the last century and still extends into ours. Radical skepticism, characterized by a belief that life and human existence lack intrinsic value and that no objective ethical truths are worth defending, has reappeared throughout history. Here is Nietzsche’s book opening: “Supposing that Truth is a woman—what then? Is there not ground for suspecting that all philosophers, in so far as they have been dogmatists, have failed to understand women—that the terrible seriousness and clumsy importunity with which they (the philosophers) have usually paid their addresses to Truth, have been unskilled and unseemly methods for winning a woman? Certainly, she has never allowed herself to be won…”

The provocative metaphor places “Truth”, in all its elusive nature, at the center of Nietzsche’s critique. Truth, of course, is neither a woman nor a man; yet, our perception of it is inevitably shaped by the preconceptions and stereotypes of those who pursue it, making more or less grandiose claims to have reached it. Nietzsche suggests that, just as philosophers may have been clumsy in their pursuit of women, so too they have been clumsy in their pursuit of Truth and the elucidation of the ethical principles that should depend on Truth to guide human action and existence.

While the great majority of well-known philosophers in history are men, a notable exception is Hannah Arendt. In a taped interview (https://www.youtube.com/watch?v=dsoImQfVsO4&t=8s), Arendt explicitly denied being a philosopher, identifying instead as a historian. Yet her body of work undeniably places her among the great philosophers, as her quest for Truth is inextricably linked to the affirmation of Good with the implication—perhaps optimistically—that Evil is often only the default outcome of the failure or refusal to think critically and independently. Her investigations into history led her to explore fundamental questions about the human condition and the structure of the human mind as reflected in the titles of two of her most remarkable works, *The Human Condition* and *The Life of the Mind*.

… Evil is often only the default outcome of the failure or refusal to think critically and independently.

In her writings, Arendt frequently refers to Henri Bergson, another Jewish thinker from the early 20th century, who addressed similar questions from a biological or organic perspective. Bergson’s ideas about the continuity of time (*la durée*) and the untamable force of life (*élan vital*) shaped his vision of evolution as a creative rather than purely deterministic process, as articulated in *The Creative Evolution*. He extended this dynamic view to ethics and spirituality in *The Two Sources of Morality and Religion*, where he highlighted the tension between the initial creative and revolutionary impulses that give rise to major ethical and religious movements and the subsequent codification of these impulses into rigid norms.

Similarly, in *Beyond Good and Evil*, Nietzsche challenges the readers to move beyond the constraints of traditional morality and religion. He advocates for a bold, dynamic understanding of life and the courage to question established conventions and, as we might say, politically correct norms. Nietzsche contends that concepts of Good and Evil are relative and culturally constructed, proposing the “*Will to Power*” as a fundamental drive inherent in all living beings. He champions the ideal of the *Übermensch*, a figure who rises above conventional morality to move humanity beyond personal suffering and failures. This ideal contrasts starkly with the fragility of his own life; like the clumsy philosophers he critiques in his writings, he struggled with troubled relationships and ultimately succumbed to a tragic end, his life cut short by syphilis.

## Good and evil or right and wrong

The distinction between Good and Evil, along with the recommendation to embrace one and condemn the other, lies at the foundation of most institutionalized religions, to which many scientists are notoriously resistant. However, Dietrich Bonhoeffer’s *Ethics* begins with a striking assertion: “The first task of Christian ethics consists in annulling such knowledge.” This perspective aligns intriguingly with Nietzsche’s call to transcend this traditional dichotomy. Bonhoeffer, a prominent theologian of the early 20^th^ century, developed a unique approach to Christian ethics centered on personal active engagement with the world. His staunch opposition to Nazism led to his imprisonment and eventual execution. Bonhoeffer rooted his ethics in the historical figure of Jesus Christ, a man of humble origins who abandoned his father’s trade as a carpenter to preach and heal in the name of his *other* Father, whom he said was God. This radical claim defied accepted religious and societal norms and led to his death. The law that Jesus proclaimed rejected the principle of retaliation—“an eye for an eye”—in favor of forgiveness. On the cross, he implored his father for forgiveness of his executioners: “They do not know what they are doing.” He proposed that knowledge and mutual understanding, not vengeance, should be at the basis of the ethical discourse and guide men’s actions.

In our modern world, can the biomedical sciences also prompt for reconciliation beyond perceived notions of Good and Evil, with the reminder that “none of us knows exactly what we are doing”? Going back to the intersection between science and philosophy, another great thinker of antiquity, Aristotle, was very much interested in biology, with ~25% of his extant corpus being devoted to this topic. His approach to study biology was remarkably scientific. He emphasized the importance of observation and conducted extensive studies of animals, dissecting specimens and carefully documenting his observations. The empirical approach at the basis of many of his treatises was revolutionary for the time and set the stage for the scientific method. By the study of bird eggs at various stages of fecundation, he established fundamental principles of organ development, with the theory of *Epigenesis* correctly proposing that organs develop in a specific order. Aristotle’s biological observations led to his concept of *telos*, as natural end-result and guiding principle in the development and organization of the body plans of animals and humans. This concept strikingly aligns with our modern understanding of homeobox genes and their pivotal role in embryogenesis, including their contribution to the cortical representation of body parts, as depicted in the homunculus model.

In our modern world, can the biomedical sciences also prompt for reconciliation beyond perceived notions of Good and Evil…

Early thinkers, Aristotle included, associated the soul, mind, and thinking with sources of body heat, particularly the heart, while the brain was only thought to have a cooling function. It was Herophilus and Erasistratus, two scholars of the 3rd century BC in Alexandria, Egypt, who have been accredited for distinguishing between sensory and motor nerves and proposing that they originated from the brain, specifically from the cerebrum and spinal cord (Konstantine and Peter, 2015). They posited that nerves are channels filled with *pneuma* (spirit or breath essential for life), which provided a critical framework for the pneuma theory of Galen of Pergamon, the most famous and influential medical doctor of antiquity who lived 300 years later (129–217 CE). Galen, who is being rediscovered today, wrote extensively not only of medicine but of philosophy, especially on logic, ethics, epistemology, causation in the natural world, and philosophy of the mind. He was a strong proponent of the primary importance of direct experimental testing over the acceptance of established paradigms in medicine, a tenet supported by another medical doctor and philosopher in 9^th^-century Bagdhad, al-Rāzī, who among other things, was also responsible for the differential diagnosis between small pox and measles (Tibi, 2006).

**Figure 2:**
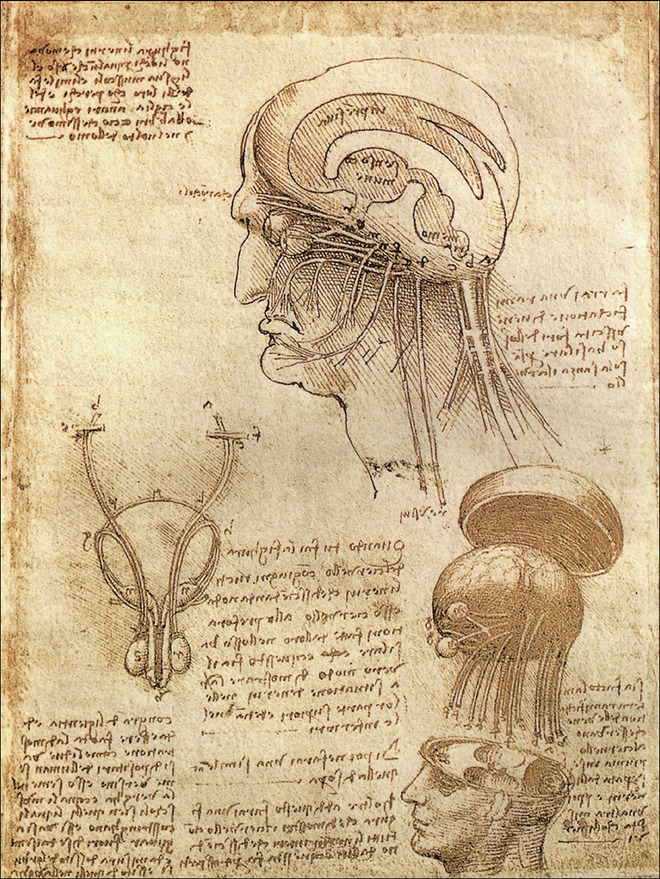
Leonardo da Vinci (1510). Studies of the head, brain and cranial nerves. Ink on parchment. Wikimedia/Public Domain.

Scientific inquiry and experimentation come with a price. Herophilus’ and Erasistratus’ seminal discoveries were based on the dissection of cadavers, a groundbreaking approach in their time, as well as the vivisection of prisoners. This is a practice that should be unfathomable today, were it not that the abuse of people—including torture and starvation to death—is still very much ongoing in many parts of the world. And yet, their observations, continued by Galen and later by the Spanish neurologist Santiago Ramón y Cajal in the early 20th century, along with studies of mental disorders, paved the way for understanding the anatomy and functions of the nervous system and the brain (Fig. 2).

Following y Cajal’s investigations of the microscopic structure of the brain in the second half of the 20th century, neurologists, biochemists and molecular biologists began to analyze the brain at the cellular, molecular and genetic level. Their work not only helped to elucidate the molecular causes of mental disorders and brain-specific diseases but also gave a more detailed insight into higher brain functions, such as memory, emotions or decision-making. We may be well on the cusp of understanding how and why we make decisions and thereby address the ancient philosophical question of why humans are capable of both: deeds of great evil and malice and deeds of great good, even sacrificing their own lives to help others. It seems though that it may be easier to understand the first rather than the second in the context of our evolutionary history.

## The neurology of making decisions

*Superior stabat lupus, inferior agnus*—Superior stands the wolf, lower stands the lamb. The oppression of others by people in stronger positions may result from the brutal dictates of evolution and natural selection: my well-being and survival depend on your demise. Intertwined with this, a perverted short-circuit of self-reward and pleasure in the brain can cause and increase the aggressiveness of small and large groups of people, armies, and nations, and their egotistical and self-absorbed leaders. Narcissistic Personality Disorder (NPD) is a complex mental health condition characterized by a pervasive sense of grandiosity, a need for admiration, and a lack of empathy for others. It was first proposed as a clinical entity for the manual of the American Psychiatric Association in 1980, which was later questioned (Jauk and Kanske, 2021). Irrespectively, studies based on neuroimaging techniques have pointed to grey-matter deficits and altered connectivity of specific brain regions (Nenadic et al, 2015; Nenadic et al, 2021) in individuals with NPD, with possible underlying alterations in the level of neurotransmitters such as serotonin and dopamine, and hormones, like cortisol and testosterone.

Experimental mouse genetic evidence indicates that not only testosterone but also estrogen and estrogen-producing neurons play a role in the aggressive behavior of both males and females (Unger et al, 2015). Studies such as these are at the basis of neuro- and behavioral endocrinology, which aims to understand the impact and interactions of hormones with brain development and function and how these affect individuals’ relation to others and their behavior (Adkins-Regan, 2009).

There is now considerable evidence for how cellular and molecular factors influence decision-making in animal model systems ranging from *C. elegans* to *Drosophila* and mice (Yapici et al, 2014). In mice, pheromones, which function as chemical mediators of interindividual behavior through modulation of neuronal function, are thought to act through the sensory activation of specific neurons in the vomeronasal organ. This activation depends on TRP2, a putative ion channel of the transient receptor potential (TRP) family that is expressed exclusively in these neurons. Male mice deficient in TRP2 expression initiate sexual and courtship behaviors toward both males and females and fail to display male-male aggression (Stowers et al, 2002; Leypold et al, 2002).

There is now considerable evidence for how cellular and molecular factors influence decision-making in animal model systems…

While human have only a vestigial vomeronasal organ and a non-functional TRP2 pseudogene (Zhang and Webb, 2003), they express several other TRP channels that can be targeted for clinical indications, including neurological and psychiatric diseases (Koivisto et al, 2021). Multifactorial genetic determinants of decision-making capability in large human populations are also indicated by GWAS studies based on several measures of risky behavior, including automobile speeding, alcohol consumption or the number of sexual partners. Many of the identified SNPs are close to genes highly expressed in the brain and with a possible role in glutamatergic and GABAergic neurotransmission (Linnér et al, 2019).

This accumulating evidence about how hormones and neurons interact raises the question to what extend are our decisions “ours” or the result of brain biochemistry? In parallel to the growing understanding of the molecular and cellular basis of decision-making, neuroimaging studies have challenged the notion of “free will”. In 1983, Benjamin Libet and his colleagues showed that a conscious decision to act was preceded by an unconscious buildup of electric activity in the brain several milliseconds before the individual “decided” to act (Libet et al, 1983). While others have confirmed Libet’s findings, his experiment has been criticized both for the setup conditions and the implied interpretation that we seem to have much less free will than we think we have.

This controversy remained until the late 2000s when John-Dylan Haynes and his research group used functional MRI (fMRI) with much higher resolution and observed a buildup of specific activity patterns in the frontopolar cortex before subjects became consciously aware of their decision (Bode and Haynes, 2009; Bode et al, 2011). However, later experiments by Haynes showed that subjects can still “veto” an unconscious decision to move until a “point of no return” about 200 ms before the actual movement takes place (Schultze-Kraft et al, 2015). While this seems to have restored the notion of ‘free will’ to some extent, these experiments are paralleled by research that merges fMRI with artificial intelligence, which allows to decode brain activity patterns before and during specific tasks, such as memory recall or decision-making—that is, to read the mind (Norman et al, 2006).

All of this raises the fundamental question of how much of our thoughts, emotions, and decisions are our own. To what extent is our ability to move beyond the constraints of traditional morality and religion, as Nietzsche and Bergson discussed, also determined by our brain chemistry and brain architecture? As John Milton wrote in *Paradise Lost* “The mind is its own place, and in itself can make a heaven of a hell, a hell of a heaven” (Fig. 1).

To what extent is our ability to move beyond the constraints of traditional morality and religion […] also determined by our brain chemistry and brain architecture?

## The greater danger: ignorance and stupidity

Beyond these molecular underpinnings, could intrinsic limitations to ‘comprehend’ be involved? Bonhoefer developed an intriguing theory of *stupidity*, arguing that it is ultimately more dangerous than evident and deliberate evil. His perspective remains as strikingly relevant today as when it was first written (*After Ten Years* in *Letters and Papers from Prison*.): “Stupidity is a more dangerous enemy of the good than malice. One may protest against evil; it can be exposed and, if need be, prevented by use of force. Evil always carries within itself the germ of its own subversion in that it leaves behind in human beings at least a sense of unease. Against stupidity, we are defenseless. Neither protests nor the use of force accomplish anything here; reasons fall on deaf ears; facts that contradict one’s prejudgment simply need not be believed—in such moments, the stupid person even becomes critical—and when facts are irrefutable, they are just pushed aside as inconsequential, as incidental. In all this, the stupid person, in contrast to the malicious one, is utterly self-satisfied and, being easily irritated, becomes dangerous by going on the attack. For that reason, greater caution is called for than with a malicious one. Never again will we try to persuade the stupid person with reasons, for it is senseless and dangerous.”

**Figure 3:**
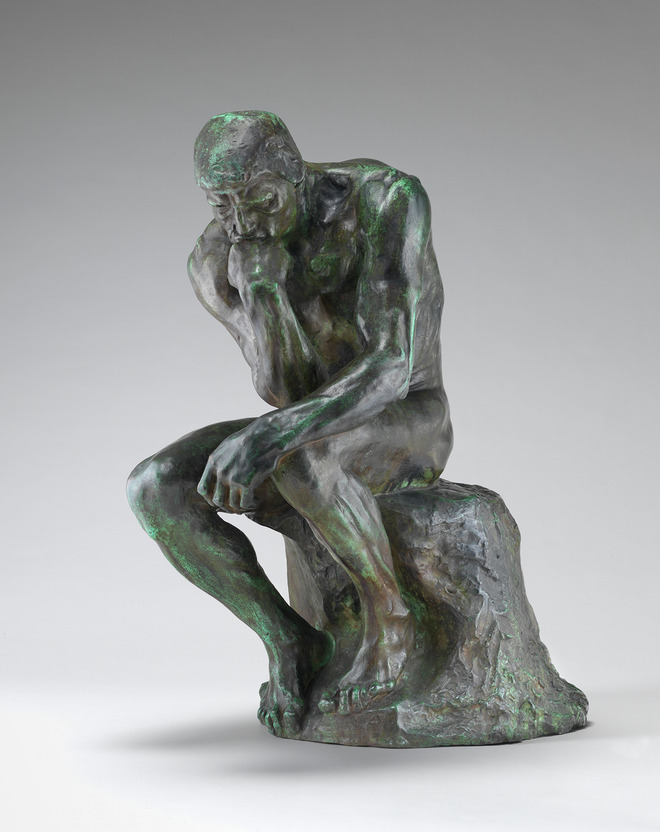
Auguste Rodin (1880). The thinker. Bronze plastic. Rawpixel/Public Domain.

In addition to the need for great caution when confronting stupidity, a particularly insidious aspect of stupidity that can affect us all is the belief that we are smarter than others. This mindset hinders mutual understanding and makes the divide between Good and Evil insurmountable. True to their title, holders of a Doctorate in *Philosophy* should be the first to recognize the limits of their own knowledge and understanding, thereby paving the way for genuine dialog with others. To start with this daunting task, and to come to some conclusion on what is the Good that we should pursue, we should first think deeply and intensively—much like Rodin’s iconic sculpture (Fig. 3).

True to their title, holders of a Doctorate in *Philosophy* should be the first to recognize the limits of their own knowledge and understanding…

## Supplementary information

Peer Review File
