# Supplementary material for: Beyond good and evil: The pursuit of philosophical and scientific truth in a time of moral ambiguity
Source: EMBO Rep. 2025 May 1;26(10):2489–93. doi: 10.1038/s44319-025-00465-x (PMC12116944; doi:10.1038/s44319-025-00465-x)
Supplement: Supplementary file 1 — Peer Review File [file 44319_2025_465_MOESM1_ESM.pdf]

EMBO reports

Beyond Good and Evil

Gian Paolo Dotto

Corresponding author(s): Gian Paolo Dotto (paolo.dotto@unil.ch)

Review Timeline:

Submission Date:	17th Apr 25
Accepted:	23rd Apr 25

Editor: Holger Breithaupt

Transaction Report: This article was editorially reviewed.
